# The Predictive Factors for Severe Leptospirosis Cases in Kedah

**DOI:** 10.3390/tropicalmed5020079

**Published:** 2020-05-14

**Authors:** Rakesh Singh Sandhu, Halim Bin Ismail, Mohd Hasni Bin Ja’afar, Sanjay Rampal

**Affiliations:** 1 Department of Community Health, Universiti Kebangsaan Malaysia, Kuala Lumpur 56000, Malaysia; halimismail@ppukm.ukm.edu.my (H.B.I.); drmhasni@ukm.edu.my (M.H.B.J.); 2 Sik District Health Office, Ministry of Health Malaysia, Sik Kedah 08200, Malaysia; 3 Centre for Epidemiology and Evidence-Based Practice, Department of Social and Preventive Medicine, Faculty of Medicine, University of Malaya, Kuala Lumpur 56000, Malaysia; srampal@ummc.edu.my

**Keywords:** leptospirosis, severe, predictive factor

## Abstract

Over the past decade, increased awareness about leptospirosis disease in developing and industrialized countries has resulted in increased numbers of leptospirosis cases being reported worldwide. About 5% to 15% of leptospirosis patients end up with severe forms of the disease. Complication due to leptospirosis requires monitoring, specific treatments, and intensive care admission, thus increasing the cost of treating severe leptospirosis cases. Currently, we have data on incident and mortality rates, but we do not have data on the number of patients with severe form of leptospirosis or how many patients have complications, and whether or not these complications were resolved. Therefore, we carried out this study to determine the predictive factors for severe leptospirosis cases in Kedah. We conducted a cross-sectional study. The data of patients diagnosed with leptospirosis were obtained from the surveillance unit, Kedah Health Department, through the e-notification system. These data were then sorted according to the hospitals where the patients were admitted. The patients’ medical records were collected, and their information was obtained using a checklist. A total of 456 confirmed leptospirosis cases were included in the study, with 199 patients classified as severe cases and 257 patients as mild cases, based on the Malaysian leptospirosis guidelines. Most patients were male (71.5%) with a mean SD age of 36.62 ± 20.75 years. The predictive factors for severe leptospirosis include abnormal lung sounds (OR: 3.07 [CI 1.58–6.00]), hepatomegaly (OR: 7.14 [1.10–45.98]), hypotension (OR: 2.16 [1.08–4.34]), leukocytosis (OR: 2.12 [1.37–3.29]), low hematocrit (OR: 2.33 [1.43–3.81]), and increased alanine aminotransferase (SGPT ALT) (OR: 2.12 [1.36–3.30]). In conclusion, knowing these predictive factors will help clinicians identify severe leptospirosis cases earlier and develop their treatment plans accordingly, to reduce the complications and death from severe leptospirosis.

## 1. Introduction

Over the past decade, increased awareness about leptospirosis disease in developing and industrialized countries has resulted in increased numbers of leptospirosis cases being reported worldwide. Incidence of leptospirosis is higher in the tropics compared to temperate countries [1,2,3]. Globally, it is difficult to determine the burden of leptospirosis disease as many countries are underreporting leptospirosis cases, and serological diagnosis of the disease is difficult. Pappas et al. [3] performed a study to determine the worldwide incidence rates for leptospirosis but were unable to evaluate the burden of leptospirosis disease as details were lacking from certain countries, such as Cambodia, Malaysia, India, Bangladesh, and Vietnam. The disease is common in developing countries and is occasionally reported in industrial countries.

Leptospirosis is a re-emerging zoonotic disease, which has a worldwide distribution and is present in all continents except Antarctica [4]. Over the past decade, numerous outbreaks have occurred, affecting thousands of people and resulting in hundreds of deaths. The human population is growing at a rapid pace and spreading to other inhabited areas, encroaching on wildlife habitats and thus increasing human and animal interactions, resulting in an increased incidence of leptospirosis cases. Leptospirosis infection was first recognized as an occupational disease among agriculture workers, people working in sewers, those who handle animals, forestry workers, people working as butchers, and outdoor workers [5,6,7,8]. A study by Lau et al. [9] showed a change in the epidemiology of leptospirosis infection, as the disease is no longer confined to these occupations only. This change may be due to the implementation of preventive measures, such as programs for rodent control, wearing protective clothing, and using detergent, which, even at a low concentration, will inhibit the survival of leptospires [10]. A study by Kariv et al. [11] in Israel showed that most leptospirosis infections are from urban areas and not from rural or agriculture areas as before. One of the reasons for this may be the migration of rural poor to cities to seek jobs and better opportunities, thus increasing the urban slump.

About 5% to 15% of leptospirosis cases result in a severe form of leptospirosis, [12,13,14] and the manifestations of severe leptospirosis include acute renal failure, acute respiratory distress syndrome, pulmonary haemorrhage, hypotension, icterus, and altered mental state [15]. A delay in diagnosing leptospirosis infection and hospitalization is associated with a poor outcome [16]. Leptospirosis mimics many other conditions, such as influenza, dengue fever, Hanta fever, rickettsioses, and other viral hemorrhagic diseases, which makes it difficult for clinicians to diagnose leptospirosis infections [17,18]. Leptospirosis patients with acute kidney injury may require dialysis, and these patients might have to do dialysis in a private center, which will be expensive, especially for lower- and middle-income groups. Hospitals, too, must bear increased costs for treating these patients. The increased numbers of leptospirosis cases over the past decade, despite the preventive and control measures in place, is worrying. It is a considerable burden for public health specialists, who must find the root cause for the rise in the number of leptospirosis cases and develop prevention and control measures. In Malaysia, very few studies have been conducted on leptospirosis, and these studies have mainly focused on the incidence, risk factors, and seroprevalence of leptospirosis. So far, there have been no studies on the predictors for severe leptospirosis in Kedah. Complications due to leptospirosis require monitoring, specific treatments, and maybe intensive care admission, thus increasing the cost of treating severe leptospirosis cases. We need to determine a method to identify these patients early, to start aggressive treatment on these patients and therefore to prevent complications and death. There are limited data on severe leptospirosis cases in Kedah. Thus, this study was conducted to identify the predictive factors for severe leptospirosis cases in Kedah.

## 2. Materials and Methods

We carried out a cross-sectional study in Kedah, which is in the northern part of peninsular Malaysia, approximately 9425 sq. km in size and with a population of around one million. Most of the community is Malay, followed by Chinese and Indian minorities. Kedah has 12 districts, and each region has rural and urban areas. Most of the people in rural areas live in villages and work as farmers, rubber tappers, or doing odd jobs. These villages have poor sanitation and garbage collection, as local municipal councils do not cover these villages. This therefore increases the risk of leptospirosis infection.

Kedah has nine hospitals and twelve District Health Offices. Data collection was conducted at nine government hospitals in Kedah from January 2014 to December 2017. Data on patients diagnosed with leptospirosis from January 2014 till December 2017 were obtained from the surveillance unit, Kedah Health Department, through the E-notification system. This is an online system, and all infectious diseases must be keyed into the system. Key information includes patient name, identification card number, age, sex, house address, occupation, name of the hospital where the patient was admitted, and MAT test results.

Demographic data such as the age, gender, ethnicity, house address, occupation, and physical and biochemical parameters of the patients with confirmed leptospirosis were obtained from the hospital records. Similarly, laboratory tests for all patients in the district and tertiary hospitals were obtained from the patient hospital records, and the data were analyzed. Patients admitted to the hospital under clinical suspicion of leptospirosis must do a rapid test for leptospirosis. Patients with positive rapid tests will then have a MAT test done for confirmation of leptospirosis infection. Confirmed leptospirosis cases included patients who had a positive rapid test and MAT titer of ≥1:400 or a 4-fold rise in the MAT titer for their paired sera. Patients with positive rapid tests and negative MAT tests were excluded from this study. Similarly, those with apositive rapid test and an equivocal MAT test were excluded from this study.

A total of 738 leptospirosis patients with positive rapid test and MAT test results were extracted from the E-notification system. Of the 738 leptospirosis patients identified, only 613 medical records of leptospirosis patients were found, and the rest could not be found when searching, or were missing. After the inclusion and exclusion criteria, only 456 complete records were obtained with all the variables and included in the study. For this study, mild cases were classified as having Influenza -like symptoms, and severe leptospirosis was defined as any confirmed leptospirosis case reported during the study period with one or more of the following criteria: jaundice, renal dysfunction, hemorrhaging, myocarditis, arrhythmia, pulmonary haemorrhage with respiratory failure, and meningitis/meningoencephalitis. This research was conducted after the approval of the ethics committee of UKM Medical Centre (UKMMC), reference number UKM PPl/111/8/JEP-2018-257 and from the Medical Research and Ethics Committee (MREC), Ministry of Health Malaysia.

Data were analyzed using Statistical Package for Social Sciences (SPSS) software version 21.0. We performed a descriptive analysis, followed by logistic regression. A multivariate analysis was carried out to determine the predictor factors for severe leptospirosis.

## 3. Results

The average age of the leptospirosis patients was 36.62 ± 20.75, and the age ranged from 2 to 97 years. The results showed that there is a lower risk of developing severe leptospirosis in patients <20 years compared to all other age groups (Table 1). Men (71.5%) were the most affected by leptospirosis infections compared to women (28.5%). The Malay race (93.4%) had the nation’s highest number of leptospirosis cases compared to non-Malays (6.6%). In this study, 76% of the patients were villagers, and 34.4%of leptospirosis patients were self-employed. Most patients had a fever lastingbetween 1–5 days (69.7%) before seeking treatment in a clinic or hospital, and were admitted to the ward for further treatment (Table 2). Additionally, some patients had a fever for more than five days (26.5%) before coming to the clinic or hospital for treatment. Most patients (n = 235, 51.5%) indicated that they had vomiting symptoms, a total of 123 patients had symptoms of diarrhea (27%), and most patients reported (n = 200, 43.9%) that they had a cough. A total of 215 leptospirosis patients complained of myalgia symptoms (47.1%), and 158 patients had arthralgia symptoms (34.6%). A total of 109 patients had symptoms of a headache (23.9%), while 87 leptospirosis patients reported abdominal symptoms (19.1%). Most patients (n = 250, 54.8%) also reported having symptoms of a reduced appetite. A chi-squared test showed a significant association between age (*p* ≤ 0.001), occupation (*p* = 0.006), fever (*p* = 0.04), and duration of fever (*p* = 0.06), with severe leptospirosis. Similarly, respiratory symptoms (*p* < 0.001), hepatomegaly (*p* = 0.005), abnormal lung sounds (*p* ≤ 0.001) and hypotension (*p* ≤ 0.001) showed a significant correlation with severe leptospirosis.

Laboratory investigations that showed a significant correlation with severe leptospirosis disease include WBC (*p* ≤ 0.001), hematocrit blood (*p* ≤ 0.001), potassium (*p* = 0.04), alanine aminotransferase tests, (*p* ≤ 0.001), and creatinine kinase tests (*p* = 0.03). Upon performing a simple logistic regression, age (*p* = 0.006), rigor (*p* = 0.04), shortness of breath (*p* ≤.001), hypotension (*p* ≤ 0.001), abnormal pulmonary sounds (*p* ≤ 0.001), and hepatomegaly (*p* = 0.01) showed a significant association with severe leptospirosis. After the odds ratio was adjusted, age (*p* = 0.006), hypotension (*p* = 0.002), abnormal pulmonary sounds (*p* ≤ 0.001), and hepatomegaly (*p* = 0.009) showed a significant association with severe leptospirosis (Table 3 and Table 4). In the final model (Table 5), the predictors for severe leptospirosis included abnormal pulmonary sounds (*p* = 0.001,OR= 3.07, 95% CI = 1.58–6.0), hepatomegaly (*p* = 0.04, OR = 7.14, 95% CI = 1.10–45.98), hypotension (*p* = 0.03, OR = 2.16, 95% CI = 1.08–4.34), WBC (*p* = 0.001, OR = 2.12, 95% CI = 1.37–3.29), hematocrit (*p* = 0.001, OR = 2.33, 95% CI = 1.43–3.81), and SGPT ALT (*p* = 0.001, OR = 2.12, 95% CI =1.36–3.30).

## 4. Discussion

This study was carried out to determine the predictive factors for severe leptospirosis in Kedah. Our findings indicate that abnormal pulmonary lung sounds, the presence of hepatomegaly, hypotension, leukocytosis, a low hematocrit value, and elevated SGPT ALT are predictors for severe leptospirosis. These signs, and the corresponding laboratory results, can be recognized following hospital admission, and the early identification of these signs and laboratory results can alert the treating physician of the possibility that the patient might end up with severe leptospirosis. In this way, aggressive treatment can be initiated from the outset. Leptospirosis is endemic to Malaysia and can present with a severe form of the disease, leading to increased morbidity and fatality. Therefore, it is of the utmost importance for us to identify the predictors for severe leptospirosis, so that we can recognize potentially fatal cases early, and carefully plan their treatment. In Kedah, we were the first to conduct a study between leptospirosis cases and severe leptospirosis.

In our study, abnormal pulmonary lung sounds, hepatomegaly, and hypotension were signs presenting a significant association with severe leptospirosis. Similar findings were also presented in a study conducted by Hochedez et al. [19] at the University of Martinique hospital from 2010 to 2013, which showed that patients with severe leptospirosis had signs and symptoms such as hypotension, abnormal chest auscultation, and jaundice. A retrospective study conducted by Smith et al. [20] on 402 leptospirosis cases in Far North Queensland observed that abnormal auscultatory findings, hypotension, oliguria, and creatinine >2 mg/dl were associated with severe leptospirosis. A study conducted by Herrmann-Storck et al. [21] among leptospirosis patients at Guadeloupe hospital found that the signs of severe leptospirosis include jaundice, abnormal pulmonary sounds, and oliguria. Similarly, a study by Pappachan et al. [22] at the University of Calicut Medical College from July to November 2002 on 282 leptospirosis patients found that signs of severe leptospirosis include nose bleeding, an unstable mental state, and tachycardia. A study conducted by Hinjoy et al. [14] in Thailand found that the signs of severe leptospirosis include conjunctival suffusion, jaundice, oliguria, chills, and calf pain. The results of these studies are different from those of our study, where conjunctival suffusion, chills and an unstable mental state showed no significant association with severe leptospirosis. Only the abnormal pulmonary sounds and hypotension are in line with our study.

A study conducted by Spichler et al. [13] in the city of Sao Paulo from January 2004 to December 2006 on 840 confirmed leptospirosis patients showed that only platelets <70,000 and creatinine >3.0 mg/dL are significantly associated with severe leptospirosis and death. The findings of their study are supported by a study conducted by Hochedez et al. [19] on 102 patients in Martinique from 2010 to 2013, where a bilirubin test >49 µmol/L, creatinine >153 µmol/L, urea >9.3 mmol/L, creatinine phosphokinase >443 U/L, lymphocytes <0.49 × 10^9^ cells/L, C- reactive protein >282 mg/L, hemoglobin <12.2 g/dL, platelets <92 × 109 cells, and prothrombin time <68% are significantly associated with severe leptospirosis patients. Similarly, a study conducted by Tubiana et al. [18] in New Caledonia from 2008 to 2011 on 71 severe leptospirosis patients showed that platelet count test results ≤50 (G/L), creatinine >200 (mM), lactate >2.5 (mM), and amylase >250 (UI/L) are closely related to the severity of leptospirosis. The findings of their study showed that patients with severe leptospirosis presenting the above test results had a poor prognosis for survival. This differs from our study, in which laboratory results such as a high WBC, low hematocrit, and elevated alanine aminotransferase showed a significant association with severe leptospirosis. Platelets did not show a significant relationship with severe leptospirosis.

Our study had several limitations. Data were obtained from the e-notification system, and hospital records. Because of this, certain clinical information was not available, such as specific laboratory investigations, signs and symptoms (these were not asked for or recorded). Our study involved nine hospitals in Kedah, and each hospital have their own test values for each laboratory test. Therefore, we were not able to present severity levels for leptospirosis.

## 5. Conclusions

Knowing the relevant factors, which can be obtained from the initial patient evaluation that are predictive of, or elevate the concern for, severe leptospirosis, will help physicians identify at-risk patients.

## Figures and Tables

**Table 1:** Socio-demographic factors for leptospirosis (n = 456).

Variables	Frequency (%)
age (years) mean ± SD	36.62 (±20.75)
Age group	
1–10	28 (6.1)
11–20	111 (24.3)
21–30	77 (16.9)
31–40	54 (11.8)
41–50	51 (11.2)
51–60	70 (15.4)
>60	65 (14.3)
Sex	
Female	130 (28.5)
Male	326 (71.5)
Race	
Malay	426 (93.4)
Non Malay	30 (6.6)
Place of living	
Village	348 (76.3)
Non village	108 (23.7)
Occupation	
Government servant	32 (7.0)
Private sector	40 (8.8)
Student	117 (25.7)
Self employed	157 (34.4)
Not working	110 (24.1)

SD: Standard Deviation.

**Table 2:** Sign and Symptoms for leptospirosis (n = 456).

Variables		Frequency (%)
Days of fever (days)	No fever	17 (3.7)
	1–5	318 (69.7)
	>5 days	121 (26.6)
Fever	No	17 (3.7)
	Yes	438 (96.1)
Vomiting	No	221 (48.5)
	Yes	235 (51.5)
Diarrhea	No	333 (73.0)
	Yes	123 (27.0)
Cough	No	256 (56.1)
	Yes	200 (43.9)
Rigor	No	386 (84.6)
	Yes	70 (15.4)
Myalgia	No	241 (52.9)
	Yes	215 (47.1)
Arthralgia	No	298 (65.4)
	Yes	158 (34.6)
Headache	No	347 (76.1)
	Yes	109 (23.9)
Abdominal pain	No	369 (80.9)
	Yes	87 (19.1)
Reduced appetite	No	206 (45.2)
	Yes	250 (54.8)
Haematuria	No	454 (99.6)
	Yes	2 (0.4)
Haemoptysis	No	445 (97.6)
	Yes	11 (2.4)
Haematemesis	No	450 (98.7)
	Yes	6 (1.3)
Epistaxis	No	451 (98.9)
	Yes	5 (1.1)
Abnormal lung sound	No	387 (84.9)
	Yes	69 (15.1)
Hepatomegaly	No	444 (97.4)
	Yes	12 (2.6)
Conjuctival suffusion	No	452 (99.1)
	Yes	4 (0.9)

**Table 3:** Association between sociodemographic factors and signs with severe leptospirosis.

Variable	Leptospirosis		OR (95%CI)	*p* Value	Adjusted OR (95% CI)	*p* Value
	Mild n	Severe n				
	(%)	(%)				
Age						
<20	94 (67.6)	45 (32.4)	Ref (1.00)	0.006	Ref (1.00)	0.006
21−60	130 (51.6)	122 (48.4)	1.96 (1.27–3.02)	0.02	1.96 (1.27–3.02)	0.02
>60	33 (50.8)	32 (49.2)	2.03 (1.11–3.70)	0.02	2.03 (1.11–3.70)	0.02
Rigors						
No	226 (58.5)	160 (41.5)	Ref (1.00)	0.03	Ref (1.00)	0.04
Yes	31 (44.3)	39 (55.7)	1.78 (1.06–2.97)	0.001	1.75 (1.03–2.96)	
Dyspnea						
No	245 (60.6)	159 (39.4)	Ref (1.00)	<0.001	Ref (1.00)	<0.001
Yes	12 (23.1)	40 (76.9)	5.14 (2.61–10.09)	<0.001	5.09 (2.59–10.03)	
Hypotension				<0.001		0.002
No	241(60.7)	156 (39.3)	Ref (1.00)		Ref (1.00)	
Yes	16 (27.1)	43 (72.9)	4.15 (2.26–7.63)		2.85 (1.49–5.46)	
Abnormal lung				<0.001		<0.001
sound						
No	242 (62.5)	145 (37.5)	Ref (1.00)		Ref (1.00)	
Yes	15 (21.7)	54 (78.3)	6.01 (3.27–11.04)		4.87 (2.59–9.13)	
Hepatomegaly				0.01		0.009
No	255 (57.4)	189 (42.6)	Ref (1.00)		Ref (1.00)	
Yes	2 (16.7)	10 (83.3)	6.75 (1.46–31.15)	0.02	7.88 (1.66–37.40)	

*p* < 0.05 CI: Confidence interval. OR: odd ratio.

**Table 4:** Association between the laboratory results and severe leptospirosis.

Variable	Leptospirosis n (%)		OR Ratio (95%CI)	*p* Value	Adjusted OR (95%CI)	*p* Value
	Mild	Severe				
WBC Normal	135 (61.9)	83 (38.1)	1.00 (ref)	<0.001	1.00 (ref)	<0.001
Low	38 (95.0)	2 (5.0)	0.09 (0.02–0.36)	0.001	0.09 (0.02–0.38)	0.001
High	84 (42.4)	114 (57.6)	2.21 (1.49–3.27)	<0.001	2.50 (1.64–3.81)	<0.001
Hematocrit						
Normal	178 (62.5)	107 (37.5)	1.00 (ref)	<0.001	1.00 (ref)	<0.001
Low	47 (37.6)	78 (62.4)	2.76 (1.79–4.26)	<0.001	2.71 (1.69–4.34)	<0.001
High	32 (69.6)	14 (30.4)	0.73 (0.37–1.43)	0.35	0.76 (0.37–1.58)	0.47
SGPT ALT						
Normal	137 (62.6)	82 (37.4)	1.00 (ref)	<0.001	1.00 (ref)	<0.001
Low	26 (78.8)	7 (21.2)	0.45 (0.19–1.08)	0.08	0.34 (0.13–0.86)	0.02
High	94 (46.1)	110 (53.9)	1.96 (1.33–2.89)	0.001	2.37 (1.54–3.63)	<0.001

SGPT ALT: alanine transaminase *p* < 0.05; CI: Confidence interval; OR: odd ratio.

**Table 5:** Association between risk factors and severe leptospirosis.

Variable	β	SE	Wald (df)	*p* Value	Corrected OR	95% CI
Abnormal lung sound	1.12	0.34	10.87 (1)	0.001	3.07	1.58–6.00
Hepatomegaly	1.97	0.95	4.27 (1)	0.04	7.14	1.10–45.98
Hypotension	0.77	0.36	4.71 (1)	0.03	2.16	1.08–4.34
WBC						
Low	−2.31	0.75	9.38 (1)	0.002	0.10	0.02–0.44
High	0.75	0.22	11.22 (1)	0.001	2.12	1.37–3.29
Hematocrit						
Low	0.85	0.25	11.37 (1)	0.001	2.33	1.43–3.81
High	−0.09	0.38	0.06 (1)	0.81	0.91	0.44–1.92
SGPT ALT						
Low	−0.81	0.48	2.85 (1)	0.09	0.45	0.17–1.14
High	0.75	0.23	10.98 (1)	0.001	2.12	1.36–3.30
Constant	−1.28	0.22	35.54 (1)	<0.001	0.28	

SGPT ALT: alanine transaminase; *p* < 0.05; CI: Confidence interval; OR: odd ratio.
